# Transparent Conducting Oxides for Photovoltaics: Manipulation of Fermi Level, Work Function and Energy Band Alignment

**DOI:** 10.3390/ma3114892

**Published:** 2010-11-02

**Authors:** Andreas Klein, Christoph Körber, André Wachau, Frank Säuberlich, Yvonne Gassenbauer, Steven P. Harvey, Diana E. Proffit, Thomas O. Mason

**Affiliations:** 1 Darmstadt University of Technology, Deptment of Materials- and Earth Sciences, Surface Science Division, Petersenstrasse 32, 64287 Darmstadt, Germany; 2 Northwestern University, Deptment of Materials Science and Engineering, 2220 Campus Drive, Evanston, IL 60208 USA

**Keywords:** transparent conducting oxides, work function, doping, band alignment, solar cells

## Abstract

Doping limits, band gaps, work functions and energy band alignments of undoped and donor-doped transparent conducting oxides ZnO, In${}_{2}$O${}_{3}$, and SnO${}_{2}$ as accessed by X-ray and ultraviolet photoelectron spectroscopy (XPS/UPS) are summarized and compared. The presented collection provides an extensive data set of technologically relevant electronic properties of photovoltaic transparent electrode materials and illustrates how these relate to the underlying defect chemistry, the dependence of surface dipoles on crystallographic orientation and/or surface termination, and Fermi level pinning.

## 1. Introduction

So-called transparent conducting oxides, or TCOs, are employed as transparent electrodes in flat-panel displays, light-emitting diodes, electrochromic windows, and solar cells [1,2,3,4]. Photovoltaic applications were recently reviewed by Fortunato *et al*. [5], where TCOs are employed as front electrodes in solar cells. Although there are important secondary requirements for such transparent electrodes (e.g., interfacial properties, chemical stability, *etc*.), the primary requirements are high electronic conductivity and good visible transparency (in thin film form). In addition, band alignment matching with the active absorber components is important. For organic conductors, the band alignment at the TCO interface is expected to depend directly on TCO work function [6,7]. For inorganic thin film solar cells, knowledge of the TCO surface potentials (e.g., ionization potential, Fermi level) provides important benchmarks for interpreting band alignment at TCO/inorganic interfaces.

Table 1 shows typical TCOs employed in several types of solar cells, adapted from Fortunato *et al*. [5], including CdTe solar cells, which commonly employ F-doped SnO${}_{2}$ front electrodes [8,9]. It can be seen that the prevailing TCOs consist of appropriately doped “basis" oxides (We refer to ZnO, In${}_{2}$O${}_{3}$, and SnO${}_{2}$ as "basis" oxides from which the more complex binary and ternary TCO compounds and/or solid solutions are derived in Zn-In-Sn-O phase space.) of ZnO (usually Al-doped AZO), In${}_{2}$O${}_{3}$ (Sn-doped ITO), and SnO${}_{2}$ (Sb-doped ATO, F-doped FTO). Although these same oxides have been employed for organic photovoltaics (OPVs), Fortunato *et al.* highlight the need for TCOs with higher work functions than are currently available [5]. For example, the HOMO levels of p-type organic absorber materials in OPVs are typically ∼5 eV or larger [10,11].

**Table 1:** TCOs employed in photovoltaic cells (after Fortunato [5]).

Cell Type	TCO	Dopants
$\left.\begin{array}{c} a-\mathrm{Si}:{\text{H}}^{§}\\ \mu \text{c}-\mathrm{Si}:{\text{H}}^{§}\\ \mathrm{HIT}-\mathrm{Si}:{\text{H}}^{§} \end{array}\right\}$	$\begin{array}{c} \mathrm{Sn}{\text{O}}_{2}\\ \text{I}{\text{n}}_{2}{\text{O}}_{3}\\ \mathrm{ZnO} \end{array}$	$\begin{array}{c} \text{F},\mathrm{Sb}\\ \mathrm{Sn}\\ \mathrm{Al},\mathrm{In},\mathrm{Ga} \end{array}$
Cu(In,Ga)Se${}_{2}$	In${}_{2}$O${}_{3}$	Sn
	ZnO	Al, In, Ga
CdTe	SnO${}_{2}$	F, Sb
	Cd${}_{2}$SnO${}_{4}$	-
Dye-sensitized Graetzel	TiO${}_{2}$*	-
	SnO${}_{2}$	F, Sb
	In${}_{2}$O${}_{3}$	Sn
OPV	high work-function${}^{‡}$	-

${}^{§}$ a-Si: amorphous silicon, *μ*c: microcrystalline, HIT: heterojunction with intrinsic layer * anatase TiO${}_{2}$ ${}^{‡}$ higher work function required than currently available [5]

We recently reported the surface potentials of sputtered TCO thin films, including the basis oxides, along with data for bulk ceramic specimens [12]. It has been demonstrated that the Fermi levels of AZO and ITO can be strongly affected by the oxygen content in the sputter gas [13,14,15,16,17,18]. In addition, independent modifications of ionization potential owing to surface dipole changes during deposition (ZnO and AZO, SnO${}_{2}$ and ATO) and/or post-deposition treatments (In${}_{2}$O${}_{3}$ and ITO) were reported. In the present work, we summarize the key factors which govern TCO behavior, namely (1) band gap, (2) Fermi level (carrier generation), (3) work function, and (4) energy level alignment at hetero-interfaces. Although we focus on the basis oxides—undoped and degenerately-doped ZnO, In${}_{2}$O${}_{3}$, and SnO${}_{2}$–our findings provide a fundamental background for understanding/controlling the behavior of more complex TCO solid solutions and compounds.

**Figure 1:**
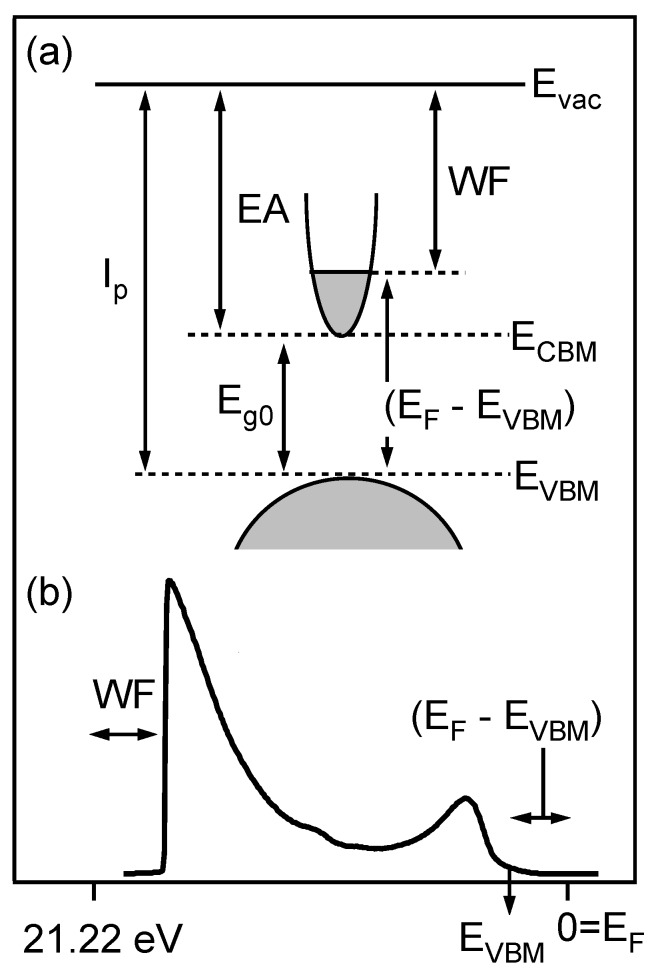
Schematics of typical TCO band structure (a) and UPS spectrum (b); $I_{p}$, WF, EA, $E_{g0}$, $E_{\mathrm{vac}}$, $E_{\mathrm{CBM}}$, $E_{\mathrm{VBM}}$, and $E_{F}$ denote ionization potential, work function (ϕ), electron affinity (*χ*), intrinsic band gap, vacuum level, conduction band minimum, valence band maximum, and Fermi level, respectively.

Figure 1a illustrates the important surface potentials and energy transitions of a generic n-type TCO. The fundamental band gap ($E_{g0}$) sets the low energy/high wavelength limit of optical transparency, which should be ≥3 eV to ensure transparency throughout the visible spectrum. On the other hand, owing to high dispersion of the conduction band, degenerate doping can result in ∼5 eV or greater additional increase in the effective band gap [19,20], shown as ($E_{F}-E_{\mathrm{VBM}}$) in Figure 1a, where $E_{F}$ is the Fermi level and $E_{\mathrm{VBM}}$ is the valence band maximum. This phenomenon is the well-known Burstein-Moss shift of the Fermi level with doping. The Fermi level ($E_{F}-E_{\mathrm{VBM}}$) therefore tells the amount of carrier doping that has been achieved. In general, the higher the Fermi level, the more conductive the TCO. Of course, at too high a doping level, free carrier absorption will shift the associated plasma frequency into the visible from the infrared and limit the optical transparency.

The work function (WF, ϕ) is essentially the Fermi level referenced to the vacuum level ($\phi =E_{\mathrm{vac}}-E_{F}$). It can be thought of as the ionization potential ($I_{p},I_{p}=E_{\mathrm{vac}}-E_{\mathrm{VBM}}$) minus the Fermi level ($E_{F}-E_{\mathrm{VBM}}$). It should be stressed that the work function is *not* a materials constant, but rather can be modified through: (1) carrier-doping, which raises the Fermi level, thereby lowering the work function (for a fixed ionization potential), and (2) modification of the surface dipole, which can increase the ionization potential and therefore the work function (for a fixed Fermi level), or a combination of these two methods. The remaining parameter in Figure 1a is the electron affinity (EA, *χ*), which is defined as ($\chi =E_{\mathrm{vac}}-E_{\mathrm{CBM}}$), where $E_{\mathrm{CBM}}$ is the conduction band minimum. The given definitions of ionization potential and electron affinity are strictly valid only for non-degenerate TCOs. We will nevertheless use the same definition also for highly doped TCOs, as the ionization potential and electron affinity provide a doping-independent measure of the surface dipole.

In the present work, the important surface potentials were measured on thin film and bulk specimens by photoelectron spectroscopy. A schematic ultraviolet (UPS) spectrum for an n-type TCO is shown in Figure 1b. The Fermi level position can be readily determined since the Fermi level of the spectrometer serves as a binding energy reference, which can be calibrated using a metallic sample. In Figure 1b, the “valence band offset" at low binding energy (on the right) corresponds to ($E_{F}-E_{\mathrm{VBM}}$) and provides a direct measure of the Fermi level at the sample surface. This offset can be measured in both UPS and X-ray photoelectron spectroscopy, XPS. At the other end of the schematic UPS spectrum the secondary electron onset, referenced to the 21.22eV Helium source energy, provides a direct measure of the specimen’s work function ($E_{\mathrm{vac}}-E_{F}$). In the present work, the Fermi levels and work functions were measured on sputter-deposited thin films of undoped (ZnO, In${}_{2}$O${}_{3}$, SnO${}_{2}$) and doped (AZO, ITO, ATO) thin films, deposited and measured *in situ* without breaking vacuum. For purposes of comparison, bulk ceramic specimens of the same materials, prepared *ex situ*, were also studied by UPS/XPS.

## 2. Results and Discussion

### 2.1. Fundamental band gaps

**Figure 2:**
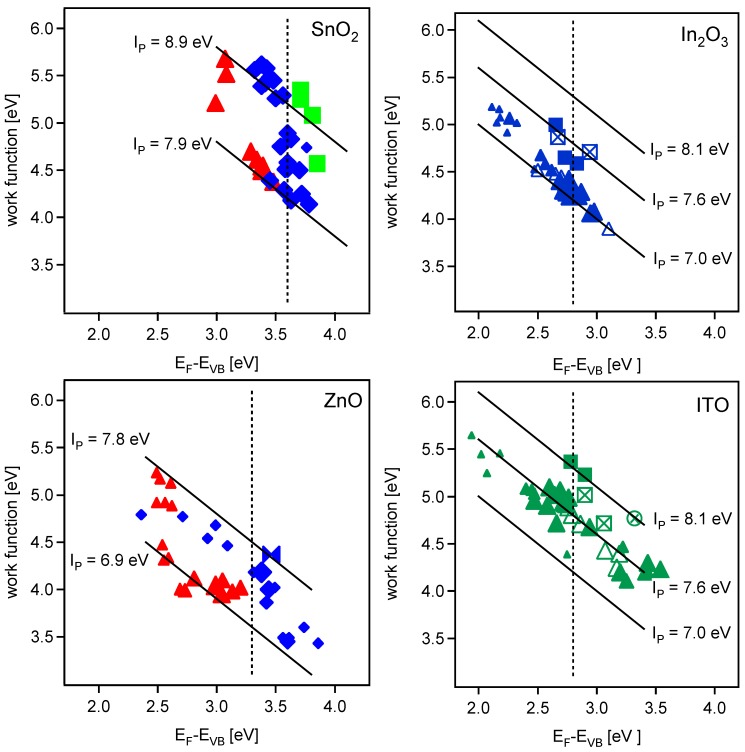
Work function *versus* Fermi level position plots. Solid lines follow constant ionization potentials, dotted vertical lines mark the intrinsic bandgap, and symbol size corresponds to substrate temperature during deposition (smallest = room temperature, largest = $400{\;}^{\circ }$C ($500{\;}^{\circ }$C for (b)). **(a)** upper left graph RF magnetron sputtered SnO${}_{2}$ (triangles) and SnO${}_{2}$:Sb (diamonds) grown at $400{\;}^{\circ }$C, and sintered ceramic SnO${}_{2}$:Sb (squares); **(b)** lower left graph DC magnetron sputtered ZnO (triangles) and ZnO:Al (diamonds) thin films, and sintered ceramic ZnO:Al (bowtie); **(c)** upper right graph In${}_{2}$O${}_{3}$ reactively evaporated (open triangles), RF magnetron sputtered (closed triangles), sintered (filled squares), and air-annealed (crossed squares); **(d)** lower right ITO sintered ceramic samples (filled squares), air-annealed thin films (crossed squares), ozone-trated thin films (crossed circle), and RF magnetron sputtered 2% doped ITO (open triangles) and 10% doped ITO (filled triangles).

A composite of all XPS/UPS-derived data is shown in Figure 2a–d as plots of work function ($E_{\mathrm{vac}}-E_{F}$) *vs*. Fermi level ($E_{F}-E_{\mathrm{VBM}}$) for each oxide system. For SnO${}_{2}$ (Figure 2a) and ZnO (Figure 2b), the data for undoped and doped oxides (ATO, AZO, respectively) have been superimposed. In the case of In${}_{2}$O${}_{3}$, for the sake of clarity, the data for undoped In${}_{2}$O${}_{3}$ (Figure 2c) and doped ITO (Figure 2d) have been plotted separately. In the case of SnO${}_{2}$ and ZnO, closed triangles represent *in situ* sputter-deposited *undoped* specimens, with relative size indicative of the substrate temperature, *i.e*., ranging from low (25 ${}^{\circ }$C) to high (500 ${}^{\circ }$C). (The data for doped films and bulk specimens are represented by closed diamonds and squares, respectively, to be discussed further below.) In the case of In${}_{2}$O${}_{3}$, closed *vs*. open triangles refer to reactively-evaporated thin films and magnetron-sputtered thin films, and in the case of ITO to magnetron sputtered films with 10 and 2 wt% SnO${}_{2}$ doping, respectively, again with relative size indicative of the substrate temperature, *i.e*., ranging from low (25 ${}^{\circ }$C) to high (400${}^{\circ }$C). (The data for bulk specimens are represented by closed squares and the crossed symbols will be discussed further below.)

Focusing on the *undoped* results for the moment, we have superimposed published values of fundamental band gap of ∼3.6 eV for SnO${}_{2}$ [21] and ∼3.3 eV for ZnO [22] on Figure 2a and b, respectively. In the case of In${}_{2}$O${}_{3}$, a band gap of ∼2.8 ± 0.2 eV (justified below) has been similarly superimposed on Figure 2c and d. It can be seen that, with the exception of a handful of In${}_{2}$O${}_{3}$ thin films, the undoped specimens (ZnO, SnO${}_{2}$, In${}_{2}$O${}_{3}$) tend to have Fermi levels less than the fundamental band gap. In other words, intrinsic defect formation (e.g., by oxygen vacancies) is insufficient to impart detectable degeneracy to the basis oxides; aliovalent donor-doping is required to shift the Fermi level significantly above the CBM (AZO, ATO, ITO). Furthermore, whereas weak metal-like Fermi edge emissions were consistently observed for *doped* films whose Fermi levels were significantly in excess of their fundamental band gaps (e.g., see Figure 4 in [17]), such emissions were only observed for In${}_{2}$O${}_{3}$ specimens with Fermi levels above the fundamental gap (∼2.9 eV), and never for undoped ZnO or SnO${}_{2}$ specimens. Therefore, the data in Figure 2 are consistent with the band gaps of 3.6 eV, 3.3 eV, and 2.8 eV for SnO${}_{2}$, ZnO, and In${}_{2}$O${}_{3}$, as shown.

The fundamental band gap of In${}_{2}$O${}_{3}$/ITO requires additional explanation and justification. It is widely known that highly degenerate thin films of ITO can exhibit band edge absorption in the range of 3.5–4.0 eV, which is much larger than the 2.8 eV fundamental band gap shown in Figure 2c and d, even accounting for Burstein-Moss shift. One possible explanation is the presence of an indirect gap that is significantly smaller than the direct band gap. However, recent density functional theory calculations indicated that the energy difference between the overall VBM and the highest occupied level at the point is less than 50 meV [23]. More recent resonant X-ray emission spectroscopy results were found to be consistent with a direct band gap for In${}_{2}$O${}_{3}$ [24]. The discrepancy has been resolved by other theoretical work [25], which showed that the separation between weak and strong optical onsets (of ∼0.85–0.9 eV) arises from the fact that transitions from the highest valence bands into the conduction band are either symmetry-forbidden or have very low dipole intensity. Therefore, the onset of strong optical absorption in degenerate ITO films occurs above 3.65–3.70 eV (the fundamental gap of 2.8eV plus the 0.85–0.90 eV separation described above). That same work set an upper limit of 2.9eV for the fundamental band gap of In${}_{2}$O${}_{3}$ [25].

**Figure 3:**
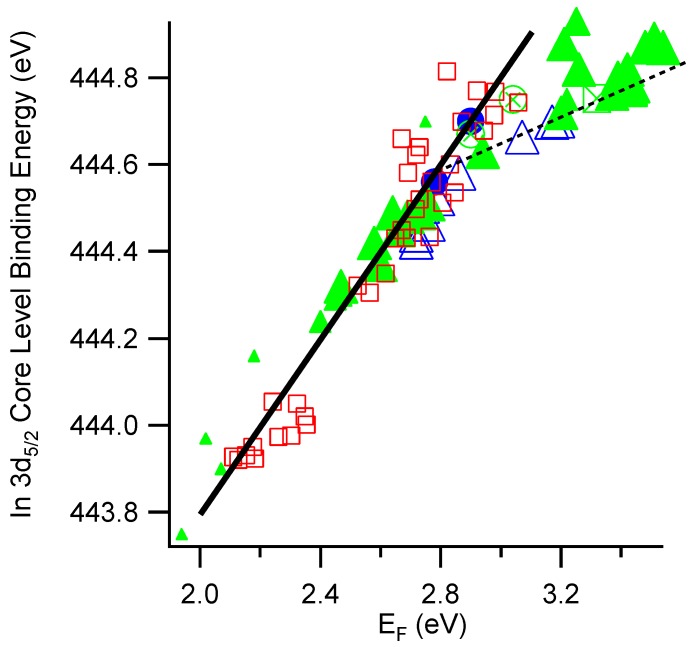
In 3d5/2 core level binding energy *vs.* Fermi level position for In${}_{2}$O${}_{3}$ and ITO. The solid line marks a slope of one. Squares represent In${}_{2}$O${}_{3}$ films and sintered ceramic samples, solid circles represent sintered ceramic ITO, solid triangles represent 10% doped ITO films, open triangles represent 2% doped ITO films, cross circles represent air-annealed ITO films (at $400{\;}^{\circ }$C), and crossed squares represent ozone treated ITO films. Symbol size reflects the substrate temperature during deposition, which ranges from room temperature (small) to $400{\;}^{\circ }$C (large).

In Figure 3 are plotted the binding energy of the In-$3d_{5/2}$ core level *vs*. the Fermi level ($E_{F}-E_{\mathrm{VBM}}$) for all the In${}_{2}$O${}_{3}$ and ITO specimens in the present work. The open squares represent undoped In${}_{2}$O${}_{3}$ (both films and sintered ceramics), the closed circles represent sintered ITO ceramics, the closed triangles 10% doped ITO films, the open triangles represent 2% doped ITO films, the cross-hatched squares represent ozone treated ITO films, and the cross-hatched circles represent ITO films annealed at 400 ${}^{\circ }$C. It can be seen that at low Fermi levels, there is a parallel change in the In-3d core level and in the Fermi level; this is indicated in Figure 3 by the solid line with slope 1. Deviation from linearity occurs in the vicinity of 2.8±0.2eV. As mentioned above, this deviation from linearity is accompanied by the appearance of metal-like emissions at the Fermi edge in UPS spectra (in specimens with Fermi levels of ∼2.9 eV or greater). Therefore, the deviation from linearity can be explained by screening of the photoemission core hole by free electrons in the conduction band [15,26,27,28]. The dashed line, representing the behavior of the degenerate specimens, roughly intersects the solid line at a Fermi level of ∼2.8 eV, which is the value we have used for the fundamental band gap of In${}_{2}$O${}_{3}$/ITO in Figure 2. Given the scatter of the data in Figure 3, we cannot at present be more precise regarding this value. Nevertheless, the data in Figure 3 provide support for the assignment of the fundamental band gap of In${}_{2}$O${}_{3}$/ITO at approximately this value.

### 2.2. Fermi level manipulation

If we consider the range of Fermi levels for donor-doped SnO${}_{2}$, ZnO, and In${}_{2}$O${}_{3}$ in Figure 2, it is clear that the behavior in ATO is markedly different from that of AZO and ITO. Whereas the Fermi levels of AZO films range from ∼2.4 eV to as high as ∼3.8 eV (Figure 2b), and the Fermi levels of ITO films range from ∼2.0 eV to ∼3.5 eV (Figure 2d), the Fermi levels of ATO films are constrained to a relatively narrow range of ∼3.3–3.75 eV (Figure 2a). Slightly larger values of Fermi level of ∼4.0 eV are obtained for fluorine doped SnO${}_{2}$, which is consistent with the higher conductivity of this material [1]. The variation of Fermi level can be understood by considering the prevailing defect models for the three materials.

**Figure 4:**
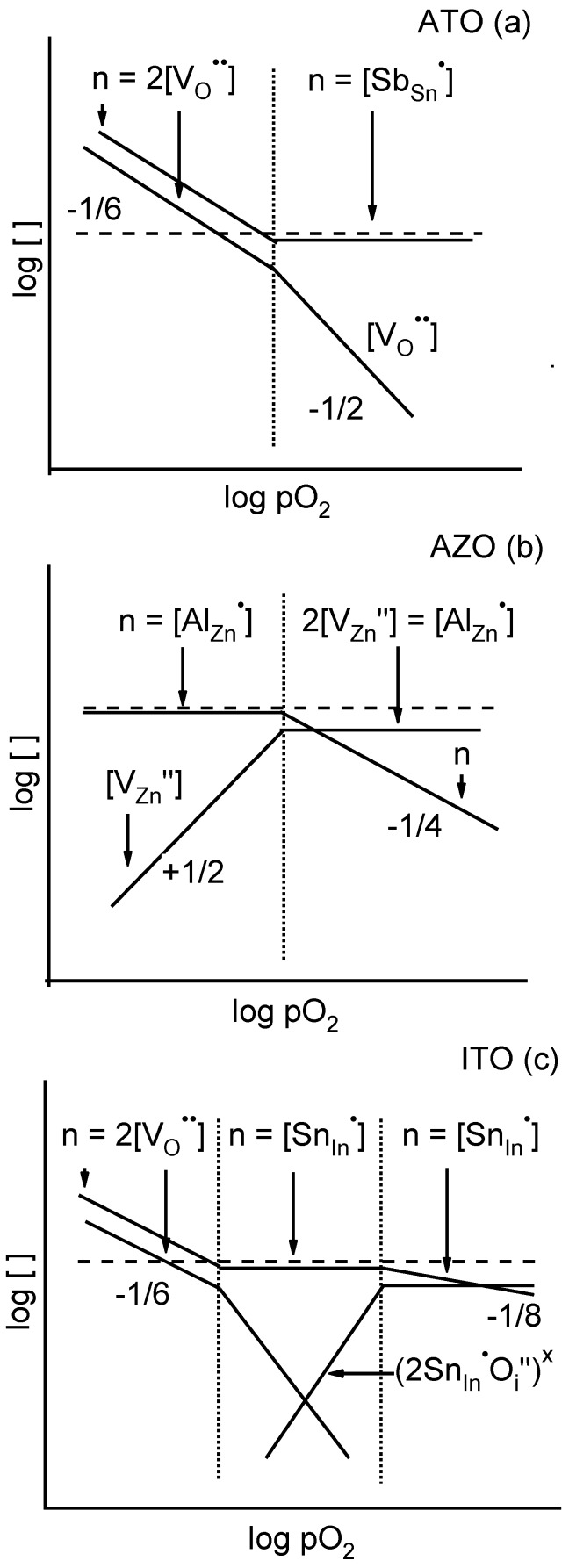
Schematic Brouwer diagrams (log concentration *versus* log pO${}_{2}$) for (a) SnO${}_{2}$:Sb (ATO), (b) ZnO:Al (AZO), and (c) In${}_{2}$O${}_{3}$:Sn (ITO). Vertical dotted lines separate different electroneutrality regimes, as denoted at the top of each column. Fractions indicate line slopes. Concentrations of electrons (*n*), doubly charged O vacancies ($\left[V_{O}^{••}\right]$), doubly charged Zn vacancies ($\left[V_{\mathrm{Zn}}^{{}^{\prime \prime }}\right]$), Sb donors ($\left[{\mathrm{Sb}}_{\mathrm{Sn}}^{•}\right]$), Al donors ($\left[{\mathrm{Al}}_{\mathrm{Zn}}^{•}\right]$), Sn donors ($\left[{\mathrm{Sn}}_{\mathrm{In}}^{•}\right]$), and Frank-Köstlin defects ($\left[{\left({\mathrm{Sn}}_{\mathrm{In}}^{•}O_{i}^{{}^{\prime \prime }}\right)}^{x}\right]$) are shown where relevant.

In Figure 4a–c are plotted the schematic Brouwer diagrams for ATO, AZO, and ITO, respectively. Brouwer diagrams are log-log plots of defect concentrations *vs.* a control variable, in this case the oxygen partial pressure. The Brouwer diagram for AZO (Figure 4b) is adapted from [29,30]. It reflects the fact that the formation of "killer" defects, in this case zinc vacancies, results in ionic compensation of the donor species (${\mathrm{Al}}_{\mathrm{Zn}}^{•}$) rather than the desired carrier generation [31,32,33,34]; the electroneutrality condition in the high-pO${}_{2}$ regime is $2\left[V_{\mathrm{Zn}}^{{}^{\prime \prime }}\right]=\left[{\mathrm{Al}}_{\mathrm{Zn}}^{•}\right]$. As pO${}_{2}$ is reduced, however, the electron population steadily rises until it becomes the prevalent species compensating the donors; the electroneutrality condition in the low-pO${}_{2}$ regime becomes $n=\left[{\mathrm{Al}}_{\mathrm{Zn}}^{•}\right]$, as shown. What this means is that the electron population in AZO can be modified by orders of magnitude through control of the pO${}_{2}$ during thin film deposition, with high oxygen contents yielding films with very small carrier contents (and Fermi levels in the band gap) and low oxygen contents yielding degenerate films (and Fermi levels above the CBM).

The Brouwer diagram for ITO in Figure 4c has been published previously [17] and agrees well with DFT calculations [29,35]. It is well known that the prevailing defect type in Sn-doped In${}_{2}$O${}_{3}$ is the so-called Frank-Köstlin (F-K) associate [36,37]. This neutral associate consists of two Sn-donors and an oxygen interstitial acceptor, or ${\left(2{\mathrm{Sn}}_{\mathrm{In}}^{•}O_{i}^{{}^{\prime \prime }}\right)}^{x}$ in Kröger-Vink notation. At high oxygen pressures, most of the Sn-donors are tied up in these neutral clusters, thereby reducing the overall electron population. Under reducing conditions, however, the interstitial oxygen can be removed from these associates, which activates the Sn-donors and increases the electron content (the middle regime in Figure 4c). Under the most reducing conditions (the leftmost regime in Figure 4c), oxygen vacancies can also be formed and further increase the electron population. What this means is that the electron population in ITO can be modified by orders of magnitude through control of the pO${}_{2}$ during thin film deposition [13,15,16,17], with high oxygen contents yielding films with very small carrier contents (and Fermi levels in the band gap) and low oxygen contents yielding degenerate films (and Fermi levels above the CBM).

In contrast, the Brouwer diagram for ATO in Figure 4a reflects the absence of either a global “killer" defect (like zinc vacancies in AZO) or a local compensating defect (like oxygen interstitials in the F-K clusters of ITO). The origin of this difference is related to the higher formation enthalpies of the possible “killer" defects, Sn-vacancies and O-interstitials, which are related to the high charge state of Sn and the rutile crystal structure of SnO${}_{2}$ [38,39]. Instead, the high-pO${}_{2}$ electroneutrality regime represents straightforward donor-doping, *i.e*., $n=\left[{\mathrm{Sb}}_{\mathrm{Sn}}^{•}\right]$, and the low-pO${}_{2}$ regime represents the possibility of additional electrons owing to intrinsic defects (e.g., oxygen vacancies). What this means is that the electron population should be high under all pO${}_{2}$ conditions, which sets ATO apart from the other two systems. This explains why there is very little change of carrier concentration with pO${}_{2}$ during deposition of the ATO films [40].

It should be noted that the observed segregation of dopant species to the surfaces of ITO (Sn) in films grown under reducing conditions [15] may also contribute to increased carrier doping of their surfaces. In other words, the low pO${}_{2}$ values during deposition yields additional carriers at the surface in two ways: (1) increased donor concentrations (owing to the segregation effect) and (2) activation of those donors (as per the Brouwer diagrams, traversing from right-to-left in Figure 4b and c).

### 2.3. Work functions

The lines superimposed on Figure 2a–d represent constant ionization potential, which is the sum of the Fermi level ($E_{F}-E_{\mathrm{VBM}}$) and the work function ($\phi =E_{\mathrm{vac}}-E_{F}$). If the ionization potential of a given oxide maintains a fixed value ($I_{p}=E_{\mathrm{vac}}-E_{\mathrm{VBM}}=\mathrm{constant}$), all the work function *vs*. Fermi level data should fall on a single line, whose slope is -1 and whose y-intercept is the ionization potential ($I_{p}$):

(1) $$\phi =-\left(E_{F}-E_{\mathrm{VBM}}\right)+I_{p}$$

In terms of the schematic diagram in Figure 1b, with a fixed ionization potential any shift in Fermi level will result in an equal and opposite change of work function; we are simply moving the Fermi level up or down between the VBM and the vacuum level ($E_{\mathrm{vac}}$).

From the data plotted in Figure 2, however, it is apparent that the ionization potentials of the oxides studied are not constants, but rather can be modified during deposition and/or by post-deposition treatments (e.g., ITO, see below). In our previous work we discussed at length the potential origins of surface dipole modifications for the three oxide systems under consideration [12]. What follows is a brief discussion of these factors, system-by-system, to provide a framework for our discussion of how the work functions of these important TCOs can be manipulated to advantage.

In the case of ATO (Sb-doped SnO${}_{2}$), the data in Figure 2a do not follow the trend of Equation 1, *i.e.*, they fall between lines rather than on a single line of constant $I_{p}$. As mentioned in the previous section, for ATO there is little or no variation of Fermi level with oxygen content employed during sputter deposition [40]. This is explained by the defect model in Figure 4a; the absence of "killer" defects in SnO${}_{2}$ accounts for the small overall changes in doping level with oxygen content during deposition. On the other hand, there are large (∼1.0 eV) changes in ionization potential (and work function) with oxygen content during deposition. In Figure 4a, the data on the $I_{p}=8.9\;$eV line were achieved for deposition under oxidizing conditions, whereas the data on the $I_{p}=7.9\;$eV line were achieved for deposition under reducing conditions, with a gradual transition for intermediate pO${}_{2}$ values. In our prior work, this was attributed to changes in surface orientation (texture) and/or surface termination [12]. For example, prior work on SnO${}_{2}$(101) surfaces yielded work functions of ∼5.7 eV after oxygen annealing *vs*. ∼4.7 eV after reduction annealing [41]. The variation of work function (∼1 eV) was attributed to different terminations of the (101) surface. Whereas oxygen annealing produces a stoichiometric surface, reduction annealing yields Sn in the Sn${}^{2+}$ oxidation state, owing to the removal of bridging and in-plane oxygens [41,42,43]. Comparable swings of work function (oxidized *vs.* reduced) have been reported for the SnO${}_{2}$(110) surface, again attributed to changes in surface terminations [44,45,46]. On the other hand, an increase in ionization potential has also been linked with a change of crystallographic orientation from (101) to (110) [47]. Therefore, an influence of surface orientation on the work function cannot be ruled out. Regardless of mechanism (change of surface orientation and/or surface termination), the ionization potential (and work function) of SnO${}_{2}$-based TCOs can be significantly modified by as much as 1.0eV by control of the oxygen content during sputter deposition. It should be noted that the data for sintered ceramic specimens tend toward the higher ionization potential ($I_{p}=8.9\;$eV) line. This is to be expected, since the specimens were sintered in air (*i.e.*, oxidizing conditions).

In contrast with ATO, the data for Al-doped ZnO (AZO) in Figure 2b more closely approximate a line (or band) of constant ionization potential, *i.e.*, they roughly behave according to Equation 1. This means that the changes in work function essentially reflect the corresponding shift of the Fermi level with doping. As discussed in the previous section, the electron population can be altered over orders of magnitude by controlling the oxygen content during sputter deposition. In Figure 2b, films grown under oxidizing conditions exhibit small Fermi levels (high work functions) and those grown under reducing conditions exhibit the opposite (high Fermi levels, low work functions). This behavior is consistent with the Brouwer diagram of Figure 4b, with redox conditions during deposition controlling the overall carrier content.

In addition, however, there are subtle changes in ionization potential for AZO films processed under oxidizing *vs*. reducing conditions. For example, it can be observed in Figure 2b that the ZnO/AZO data are bracketed by two lines of constant ionization potential. Although the effect is not as pronounced as with the SnO${}_{2}$/ATO films, AZO films deposited under oxidizing conditions tend to approach the $I_{p}=7.8\;$eV line (at small Fermi levels), whereas those grown under reducing conditions tend to approach the $I_{p}=6.9\;$eV line (at large Fermi levels). The significance of these two lines is that the lower line ($I_{p}=6.9\;$eV) has been established for the polar, zinc-terminated (0001) surface of ZnO, whereas the upper line ($I_{p}=7.8\;$eV) has been established for the polar oxygen-terminated $\left(000\bar{1}\right)$ surface and the nonpolar $\left(10\bar{1}0\right)$ surface of ZnO [48,49,50]. These results suggest that processing conditions not only alter the carrier content (Fermi level), but also the surface dipole (and therefore the ionization potential). It should be noted that the datum for a sintered ceramic specimen falls near the higher ionization potential ($I_{p}=7.8\;$eV) line. Since large ionization potentials are seen for all orientations except (0001) [51], we conclude that the ceramic specimens are dominated by orientations other than (0001).

There is one significant difference between the AZO behavior and that of the other two systems. Whereas the surface dipole of either ATO or ITO can be modified by post-deposition treatments (*i.e.*, by ozone treatments and/or oxidation at intermediate temperatures), the ionization potential of AZO films is fixed during deposition and appears to be unchangeable. This is to be expected, since a change of surface dipole requires a change of grain orientation, which is virtually impossible to achieve by post-deposition treatments.

The work function *vs.* Fermi level data for thin film ITO (Figure 2d) closely follows Equation 1, *i.e.*, at roughly constant ionization potential ($I_{p}=E_{\mathrm{vac}}-E_{\mathrm{VBM}}=7.6\;$eV). This is consistent with prior studies [14,15,17,52]. The movement right or left along this line indicates a shift of the Fermi level between more or less constant values of $E_{\mathrm{VBM}}$ and the vacuum level (see Figure 1b). The large range of achievable work functions (∼4.2 to ∼5.6 eV) derives from the correspondingly large range of achievable Fermi level positions, as discussed in terms of the Brouwer diagram (Figure 4c) in the prior section. It should be stressed that the larger values of work function along this line (to the left of the fundamental band gap) are not desirable, in that films with Fermi levels much below the fundamental band gap are not degenerately doped, *i.e.*, they have low conductivities and therefore are not suitable as TCOs.

We do not comment further about the behavior of undoped In${}_{2}$O${}_{3}$ films (Figure 2c), given that these films are insufficiently doped/conductive for TCO applications, other than to note that the data fall on a line of lesser ionization potential ($I_{p}\approx 7.0\;$eV). This may be attributable to the absence of Sn, which when present seems to be associated with higher ionization potentials, *i.e.*, $I_{p}$(In${}_{2}$O${}_{3}$)$<I_{p}$(ITO)$<I_{p}$(SnO${}_{2}$/ATO).

What is noteworthy about the ITO films is the ability to modify their ionization potentials/work functions through post-deposition treatments. For example, the crossed squares in Figure 2d represent thin film specimens, whose original data lie on the $I_{p}=7.6\;$eV line, which have subsequently been air-annealed at $400^{\circ }$C. There are insignificant changes in Fermi level, but as can be seen, their work functions (and ionization potentials) increase significantly. Similarly, the crossed circle represents an ITO film, originally lying on the $I_{p}=7.6\;$eV line, which was subsequently subjected to ozone treatment at room temperature. This datum falls on the upper line of $I_{p}\approx 8.1\;$eV, which is also where the data for sintered ceramic specimens lie.

The explanations for the post-deposition modifications of ionization potential in ITO films are not as compelling as for the SnO${}_{2}$-based and ZnO-based films, owing to a lack of experimental and theoretical studies of specific crystallographic orientations/terminations. Plausible explanations for the origin of the observed surface dipole changes in In${}_{2}$O${}_{3}$-based materials include modification of the surface dipole through change of surface terminations (as in the case of SnO${}_{2}$) and/or possibly through the incorporation/implantation of surface species during oxidation or ozone treatments. The bixbyite structure of In${}_{2}$O${}_{3}$ is unique in its abundance of "structural oxygen vacancies" (*i.e.*, oxygen interstitial positions) [37], which can accommodate additional species, whether in the bulk (see Figure 4c) or in the surface. Regardless of explanation, it is well established that post-deposition oxidation treatments can be employed to increase the work function of ITO-based TCOs [53,54,55,56,57].

### 2.4. Energy band alignment

The energy band alignment is another important factor in determining electrical properties of heterojunction devices [58]. It is here necessary to distinguish between TCO contacts to organic and to inorganic semiconductors. The energy band alignment with organic materials is largely governed by alignment of the vacuum level, so the work function of the TCO determines the energy barriers at the contacts [6,7]. In that case, modification of band alignment can be obtained following the general dependencies outlined in the previous section. In contrast, the presence of intrinsic and extrinsic interface states at interfaces between inorganic materials lead to interface dipoles, which can result in large deviations from the vacuum level alignment [59,60]. Furthermore, TCO interfaces in thin film solar cells are polycrystalline and the TCO and its contact partner have different crystal structures and/or different lattice constants. To date, no general understanding of such interfaces exists.

The almost exclusively used contact partner for the TCO in thin film solar cells with CdTe and Cu(In,Ga)(S,Se)${}_{2}$ absorber materials is CdS [61]. The main role of this interface is the collection of electrons, which is achieved by bringing the Fermi level close to the conduction band. This is, however, not sufficient for high efficiency. Neither in CdTe nor in Cu(In,Ga)(S,Se)${}_{2}$ thin film solar cells are high efficiencies obtained by direct contact of the absorber (CdTe or Cu(In,Ga)(S,Se)${}_{2}$) with the TCO, although the condition of a Fermi level close to the absorber conduction band can be fulfilled. The insertion of the CdS “buffer" layer most likely results in a strongly reduced minority carrier recombination at the absorber/buffer interface, compared to the absorber/TCO interface.

Several studies related to TCO/CdS interfaces employing stepwise evaporation of CdS onto TCO and stepwise sputter deposition of TCOs onto CdS, as well as sputter depth profiles, have been performed [62,63,64,65,66,67,68]. An extensive investigation of the CdS/ZnO interface is described in [18]. It turns out that the band alignment can depend significantly on processing, which is mainly related to two effects: (1) due to the dissimilar structure of the TCO and CdS, amorphous phases may occur at the interface; (2) the Fermi level in CdS seems to be restricted to a range 1.8–2.2 eV above the valence band maximum. This Fermi level pinning is particularly important as the Fermi level in TCOs can vary by as much as 1 eV (see Section 2.2), which can consequently lead to an apparent dependence of band alignment on doping. The effect is most pronounced at interfaces between ZnO and In${}_{2}$S${}_{3}$, an alternative buffer layer material for Cu(In,Ga)(S,Se)${}_{2}$ thin film solar cells [18,60].

**Figure 5:**
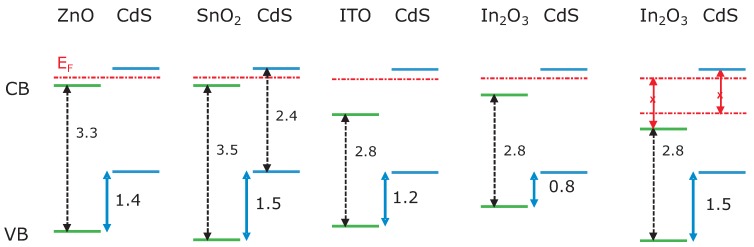
Energy band alignment at TCO/CdS interfaces as determined from photoelectron spectroscopy using stepwise deposition experiments and sputter depth profiles. All values are given in electronvolts. The first four plots show the interpretation of experimental data, whereas the last depicts why it is not possible to find a Fermi level position consistent with the allowed Fermi level range in In${}_{2}$O${}_{3}$ and the 1.8-2.2eV range found for CdS. In consequence, a local dipole occurs at the interface, shifting the energy bands of In${}_{2}$O${}_{3}$ upwards as shown in the experimental In${}_{2}$O${}_{3}$/CdS band diagram.

A summary of experimentally determined energy band alignments at TCO/CdS interfaces is given in Figure 5. For the CdS/ZnO interface we have chosen a valence band offset of 1.4eV. This value is consistently derived from CdS deposition onto both undoped and Al-doped ZnO, indicating that Fermi level pinning is not affecting the alignment [18]. Since the valence bands of the three basis TCOs are derived mainly from O 2p orbitals, it is expected that the valence bands are at comparable energy and that the valence band offsets between the TCOs and CdS are comparable. This expectation is fulfilled for ZnO and SnO${}_{2}$, but the valence band offsets between CdS and ITO and In${}_{2}$O${}_{3}$ are considerably smaller than those between CdS and ZnO and SnO${}_{2}$. The deviation can again be attributed to the Fermi level pinning in CdS and to the smaller energy gap of In${}_{2}$O${}_{3}$. Assuming a valence band energy for In${}_{2}$O${}_{3}$ similar to those of SnO${}_{2}$ and ZnO as done in the final graph of Figure 5, it is not possible to find a Fermi level position consistent with the allowed range in In${}_{2}$O${}_{3}$ (see above) and the 1.8–2.2 eV range found for CdS. In consequence, a local dipole occurs at the interface, shifting the energy bands of In${}_{2}$O${}_{3}$ upwards. An interface experiment between SnO${}_{2}$ and In${}_{2}$O${}_{3}$ has independently verified their similar valence band energies and this interpretation of measurements. One can thus conclude that valence band offsets between the three basis TCOs and any inorganic semiconductor are generically of similar magnitude, if they comply with the possible range of Fermi levels.

### 2.5. Practical ramifications

**Figure 6:**
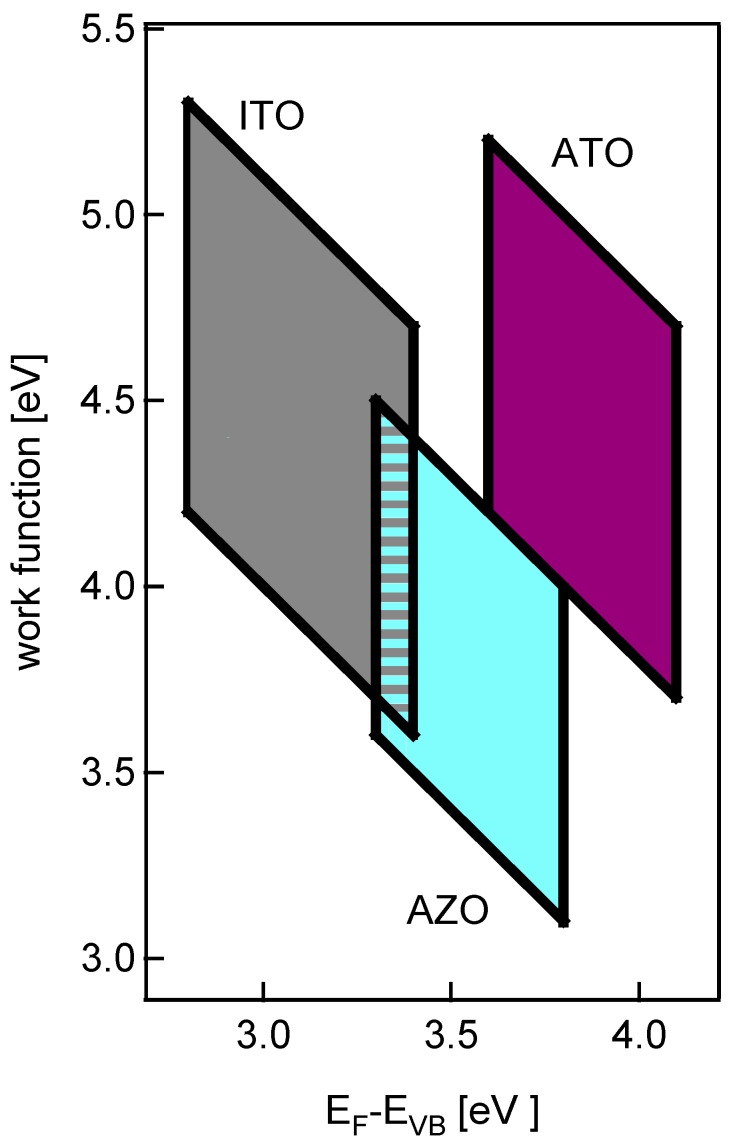
Work function *versus* Fermi level position for ATO, AZO, and ITO. The left side of each parallelogram corresponds to the bandgap, the right side corresponds to the maximum Burstein-Moss shift, and the top and bottom lines correspond to known ranges in ionization potential.

Key parameters of interest to photovoltaic applications of TCOs include high electronic conductivity, good visible transparency (in thin film form), work function and energy band alignment. These properties can be judged on the basis of the modified work function *vs.* Fermi level diagram shown in Figure 6. In this diagram, TCO-appropriate properties are represented as shaded parallelogram boxes for each oxide. The left (low Fermi level) side of each box corresponds to the fundamental band gap of the corresponding oxide. A fundamental band gap of 3.1eV or greater is a requisite for full optical transparency. (As discussed previously, In${}_{2}$O${}_{3}$ and ITO retain full optical transparency in spite of having a smaller fundamental band gap (∼2.8 eV), owing to weak (or symmetry-forbidden) transitions from the top of the valence band.) The left side of each parallelogram is important for another reason, since it represents the onset of significant electronic conductivity (through degenerate doping). It further represents an estimate for the conduction band alignment at interfaces between TCOs and inorganic semiconductors, as it can be assumed that the valence band maxima of the TCOs are at comparable energy. As SnO${}_{2}$ exhibits the highest conduction band energy, it may in principle provide the best electrical contact to n-type conductors. It is recalled however, that this is only true if strong interfacial dipoles and/or Fermi level pinning are not present [18,60]. The right side of each box represents a generous Burstein-Moss shift of Fermi level by doping to ∼0.5 eV above the fundamental band gap [19,20]. (The right boundary is 0.6eV above the fundamental gap in the case of ITO, to account for the larger Fermi levels observed in thin films.) The top and bottom of each box represent the potential modifications in surface dipole found in the present work by control of the oxygen content during film deposition. In the case of SnO${}_{2}$ and ZnO, however, the upper and lower limits are consistent with work function variations in the literature for specific orientations and/or surface terminations. In addition, the upper limits in each instance are consistent with the data for sintered ceramic specimens, which can be expected to represent relaxed, fully-oxidized surfaces.

The boxes in Figure 6 are therefore reasonable representations of the Fermi levels and work functions available for each type of TCO. For ATO, the range of work functions is from 3.8eV to 5.2eV. For AZO, the range of work functions is significantly lower, from 3.1eV to 4.5eV. And for ITO, the range of work functions is from 3.6eV to 5.3eV. As mentioned previously, the ionization potentials (work functions) of both ATO and ITO films can be significantly increased by post-deposition treatments (ozone, oxidation at intermediate temperatures), but never above the values indicated by the boxes in Figure 6. It should be stressed that changes in “effective" work function, over and above the “intrinsic" (oxygen-related) modifications in the present work, can be achieved by surface chemical treatments as pointed out by Armstrong *et al*. [69].

## 3. Conclusions

Photoelectron spectroscopy (XPS, UPS) data were collected for sputtered and reactively-evaporated thin films of the undoped “basis" oxides (ZnO, In${}_{2}$O${}_{3}$, SnO${}_{2}$) and their donor-doped counterparts (Al-doped AZO, Sn-doped ITO, Sb-doped ATO) to compare with data for air-sintered ceramic specimens. When these data are plotted as work function *vs.* Fermi level (relative to the VBM), these plots provide important information regarding the fundamental band gaps, the carrier contents, and the work functions/ionization potentials of these important TCO systems.

It was observed that the data for the undoped oxides tend to terminate at or around the fundamental band gaps of the host oxide in question: ∼3.3 eV for ZnO, ∼2.8 eV for In${}_{2}$O${}_{3}$, and ∼3.6 eV for SnO${}_{2}$. Extrinsic doping is required to significantly donor-dope these oxides, raising the Fermi levels as much as 0.5 eV above the fundamental band gap, owing to the well known Burstein-Moss shift. It should be noted that In${}_{2}$O${}_{3}$ and ITO retain full optical transparency in spite of having a smaller fundamental band gap (2.8 eV), owing to weak (or symmetry-forbidden) transitions from the top of the valence band.

Whereas wide excursions in Fermi level were observed for AZO and ITO thin films, the ATO data fall in a relatively narrow range. These behaviors are consistent with the corresponding Brouwer diagrams (defect population *vs.* pO${}_{2}$) of the three oxides. AZO exhibits ionic compensation of donors, *i.e.*, it possesses a “killer" defect (zinc vacancies). Only under reducing conditions is there a transition from a purely ionic regime ($2\left[V_{\mathrm{Zn}}^{{}^{\prime \prime }}\right]=\left[{\mathrm{Al}}_{\mathrm{Zn}}^{•}\right]$) to the desired electronic regime ($n=\left[{\mathrm{Al}}_{\mathrm{Zn}}^{•}\right]$). As a result, the electron population can be varied over a wide range as the pO${}_{2}$ is varied during deposition. In ITO, the existence of a neutral associate reduces the concentration of charged donor species under high pO${}_{2}$ deposition conditions. Under reducing conditions, oxygen interstitials are removed from the associates, activating the Sn-donor species. Therefore, the electron population (and Fermi level) can be varied over a wide range as the pO${}_{2}$ is varied during deposition. In contrast, ATO appears to lack either a global “killer" defect or a local (neutral) defect associate. Electron contents (and Fermi levels) are therefore relatively insensitive to pO${}_{2}$ during deposition.

All three oxides exhibit a range of ionization potentials (and therefore work functions). In ATO the relatively large ∼1.0 eV excursions can be related to changes of surface dipole associated with modifications of surface termination (oxidation state) and/or crystallographic orientation. In AZO similar ∼0.9 eV excursions can be related to changes of surface dipole associated with modifications of crystallographic orientation, which cannot be modified by post-deposition treatments. The origins of the smaller ∼0.6 eV excursions in ITO are unclear at the present time. For practical applications, however, it is relatively easy to modify the surface dipole of ITO by post-deposition treatments (e.g., with ozone- or other oxidation-treatment at intermediate temperature).

It is possible to map out technologically useful ranges on work function *vs.* Fermi level plots for transparent electrode applications, including photovoltaics. Parallelogram-shaped boxes can be identified, bounded on the low Fermi level end by the fundamental band gap (onset of suitable doping/conductivity) and on the high Fermi level by the Burstein-Moss shift (fundamental band gap plus ∼0.5 eV, or slightly larger for ITO). The upper and lower bounds are defined by lines of constant ionization potential associated with documented surface dipole changes owing to surface orientation and/or termination differences. The diagrams also allow for estimates of band alignment at interfaces. While the functions are relevant for interfaces with organic semiconductors, the valence bands of all three basis TCOs in contact with inorganic semiconductors are at comparable energy resulting in comparable valence band offsets. The variation of Fermi level with respect to the valence band maximum therefore directly corresponds to the variation of Fermi level position at the interface, if it is not otherwise restricted by materials limitations.

## 4. Experimental Section

Thin films of undoped and doped TCOs used throughout this study were prepared by DC (ZnO-based) or RF (SnO${}_{2}$- and In${}_{2}$O${}_{3}$-based) magnetron sputtering from 2-inch diameter ceramic targets. The sputtering conditions were 5–25 W, target-to-substrate distance of 5–10 cm, and 0.5–5 Pa pressures of Ar/O${}_{2}$ gas mixtures with oxygen contents of 0–50%. The substrate temperature was varied from 25–500 °C Glass substrates with thin coatings of F-doped SnO${}_{2}$ were used for UPS measurements to avoid charging problems in less conducting films. The targets employed for doped films were 2 wt% Al-doped ZnO, 3 wt% Sb${}_{2}$O${}_{5}$-doped SnO${}_{2}$, and 2 wt% or 10 wt% SnO${}_{2}$-doped In${}_{2}$O${}_{3}$. Targets purchased from different sources (Lesker, Mateck), described more fully elsewhere [13,14,16,17,18,53], had purity levels of 99.9% or better. X-ray diffraction was employed to examine the crystallographic structure of select samples, for which all observed reflections could be attributed to wurtzite (ZnO), bixbyite (In${}_{2}$O${}_{3}$), or rutile (SnO${}_{2}$) [18,47,70].

Additional films of In${}_{2}$O${}_{3}$ were deposited using reactive evaporation of indium metal under pO${}_{2}$ conditions varying from $10^{-6}$ to $10^{-3}\;$mbar on various substrates at a substrate temperature of $220{\;}^{\circ }$C. Details of these films, their deposition conditions, and their surface and interface characterization are given in [52,71,72].

Bulk polycrystalline specimens were prepared by conventional solid state reaction at high temperature (1250–1350 °C) with repeated grindings/firings to achieve phase-pure products. Polymer gloves were employed for handling at all stages to avoid contamination of powders/pellets. High purity reagents (10–100 ppm total metal impurities) were weighed to appropriate stoichiometry and thoroughly mixed under acetone with agate mortar and pestle. Disc-shaped specimens were uniaxially cold-pressed to 150–200 MPa. Each pellet was surrounded by a bed of sacrificial powder of identical composition to eliminate contact with the alumina crucibles employed and to reduce volatilization (especially zinc) from the samples during firing. Each crucible was nested within a series of larger crucibles with lids to also discourage volatilization. Overall weight losses (including the sacrificial powders) never exceeded 1 percent.

Photoelectron spectroscopy measurements were almost exclusively carried out in the DArmstadt Integrated System for MATerials research (DAISY-MAT) on a Physical Electronics PHI 5700 spectrometer [73]. This system incorporates thin film preparation chambers with multi-technique surface analysis capabilities in an ultrahigh vacuum environment. All thin film specimens were deposited, transferred, and measured without breaking vacuum, thereby avoiding typical hydrocarbon and water adsorbates. XPS measurements were carried out with monochromatic Al-K*α* radiation (hν=1,486.6eV). With a 5.85eV pass energy, the system yields an overall experimental resolution of better than 400meV, as determined by the Gaussian broadening of the Fermi edge of a clean Ag specimen. With such energy resolution, binding energies of core levels and valence band maxima can be determined with an accuracy of ∼0.02 and ∼0.05 eV, respectively. The spectrometer was calibrated at regular intervals with sputter-cleaned reference specimens (Cu, Ag, Au). All XPS spectra showed only emission of elements anticipated from the target compositions. In the case of ATO, there were negligibly small deviations from the nominal composition [40]. However, with ITO there was detectable segregation of Sn to the surface for films made under reducing conditions (as much as a factor of 1.5 [15]), and for AZO there was significant Al enrichment when films were deposited at higher substrate temperatures (as much as a factor of 2 or 3 [18]).

UV photoelectron spectroscopy was carried out with a helium discharge lamp (hν=21.22eV) in normal emission with a sample bias of -1.5V. The positions of the valence band maxima were determined using linear extrapolation of the leading edge of the valence band emissions, and work functions were determined from the half height of the secondary electron onset, which shows a characteristic width of ∼0.2 eV.

Energy band alignments are determined by a standard procedure outlined in [33]. A clean substrate is first prepared by deposition in one of the deposition chambers of the DAISY-MAT system and analyzed using XPS/UPS. The evolution of the spectra with increasing film thickness is followed by repeated deposition/analysis cycles in a stepwise deposition experiment until the substrate lines are completely attenuated, which occurs typically after 5–10 nm of film deposition. The valence band maxima positions are extracted from the core level binding energies by subtracting reference values for the core level to valence band maximum binding energy differences. These levels are constant for a given material, unless degenerate doping leads to additional screening of the photoemission core hole (see discussion of Figure 3). Valence band offsets can then determined with a typical uncertainty of ±0.1 eV. Conduction band offsets are calculated from the valence band offsets by adding band gap values from literature.
